# Causal associations between human gut microbiota and cholelithiasis: a mendelian randomization study

**DOI:** 10.3389/fcimb.2023.1169119

**Published:** 2023-05-25

**Authors:** Wei Li, Ao Ren, Qiong Qin, Ling Zhao, Qiufeng Peng, Ruidong Ma, Shiqiao Luo

**Affiliations:** Department of Hepatobiliary Surgery, The First Affiliated Hospital of Chongqing Medical University, Chongqing, China

**Keywords:** gut microbiota, cholelithiasis, mendelian randomization, causality, genetic association

## Abstract

**Background:**

There was some evidence that gut microbiota was closely related to cholelithiasis, but the causal relationship between them remained unclear. In this study, we try to use Two-sample Mendelian randomization (MR) to clarify the potential causal relationship between gut microbiota and cholelithiasis.

**Methods:**

Summary Genome-Wide Association Studies (GWAS) statistical data for gut microbiota was obtained from MiBioGen, and the data of cholelithiasis was obtained from UK Biobank (UKB). Two-sample MR analyses were performed to assess causalities between gut microbiota and cholelithiasis mainly using the inverse-variance weighted (IVW) method. Sensitivity analyses were used to determine the robustness of the MR results. Reverse MR analyses were performed to examine the reverse causal association.

**Results:**

Our research results, based primarily on the IVW method, support the existence of a causal relationship between nine gut microbial taxa and cholelithiasis. We observed a positive association between G*enus Butyrivibrio* (p=0.032), *Genus Lachnospiraceae_UCG_001* (p=0.015), *Genus Ruminococcaceae_NK4A214_group* (p=0.003), *Genus Ruminococcaceae_UCG_011* (p=0.010) and cholelithiasis, while *Order Rhodospirillales* (p=0.031), *Genus Actinomyces* (p=0.010), *Genus Phascolarctobacterium* (p=0.036), *Genus Rikenellaceae_RC9_gutgroup* (p=0.023), *Genus Ruminococcaceae_UCG_013* (p=0.022) may be associated with a reduced risk of cholelithiasis. We did not find a reverse causal relationship between cholelithiasis and 9 specific gut microbial taxa.

**Conclusions:**

This is the first mendelian randomization study to explore the causalities between specific gut microbiota taxa and cholelithiasis, which may provide new ideas and a theoretical basis for the prevention and treatment of cholelithiasis in the future.

## 1 Introduction

Cholelithiasis is a common disease of the digestive system with a prevalence of 10-20% in the population, and it is on the rise (Lammert et al., 2016). In addition, cholelithiasis is an important risk factor for gallbladder cancer (Mhatre et al., 2021), raising the economic burden on society, it has become a public health issue of increasing concern (Lammert et al., 2016). Cholelithiasis is closely linked to genetic factors and is also influenced by non-genetic risk factors such as metabolic disorders (Lammert et al., 2016).

The gut microbiota is closely related to body metabolism, immune regulation and the stability of the intestinal mucosal barrier (Hitch et al., 2022), and human gut microbiota is an important component of the intestinal microbial system (Jandhyala et al., 2015). The healthy gut microbiota is predominantly constituted by the *Phyla Firmicutes*, *Phyla Bacteroidetes*, *Phyla Actinobacteria* and *Phyla Verrucomicrobia* (Jandhyala et al., 2015). Recent studies have reported the close association of gut microbiota with a variety of diseases, including cholelithiasis (Lammert et al., 2016). The earliest studies on bacteria and gallbladder stones date back to the 1960s, Maki et al. first demonstrated that the bacteria in the gallbladder can produce *β-glucuronidase* (β-GD), and *β-glucuronidase* plays an important role in the formation mechanism of gallstones (Maki, 1966). In recent years, we have learned more about the relationship between gut microbiota and cholelithiasis using high-throughput sequencing, and Wu et al. were the first to show that gallbladder stone formation was associated with intestinal flora disorders using 16SrRNA sequencing in patients (Wu et al., 2013). Most of the current studies are observational studies with limited sample sizes and influenced by confounding factors, the results can show that gut microbiota is associated with cholelithiasis, but it cannot reveal a specific cause-and-effect relationship between them.

Mendelian randomization (MR) methods use single nucleotide polymorphism (SNP) as an instrumental variable (IVs) to assess the causal relationship between exposure and outcome (Emdin et al., 2017). In contrast to observational studies, MR uses random segregation in allelic inheritance to avoid confounding factors and reverse causality on study outcomes, achieving a study design similar to that of randomized controlled studies (Emdin et al., 2017; Bowden and Holmes, 2019). Several studies have used a mendelian randomization method to assess the potential causal relationship between gut microbiota and diseases (Xu et al., 2021; Chen et al., 2022; Lee, 2022). In this study, we performed the first two-sample MR analysis of the Genome-Wide Association Studies (GWAS) summary data containing gut microbiota and cholelithiasis, revealed the causal impact of gut microbiota on cholelithiasis, provided new biomarkers for the clinical management of cholelithiasis.

## 2 Methods

### 2.1 Study design

**Figure 1:**
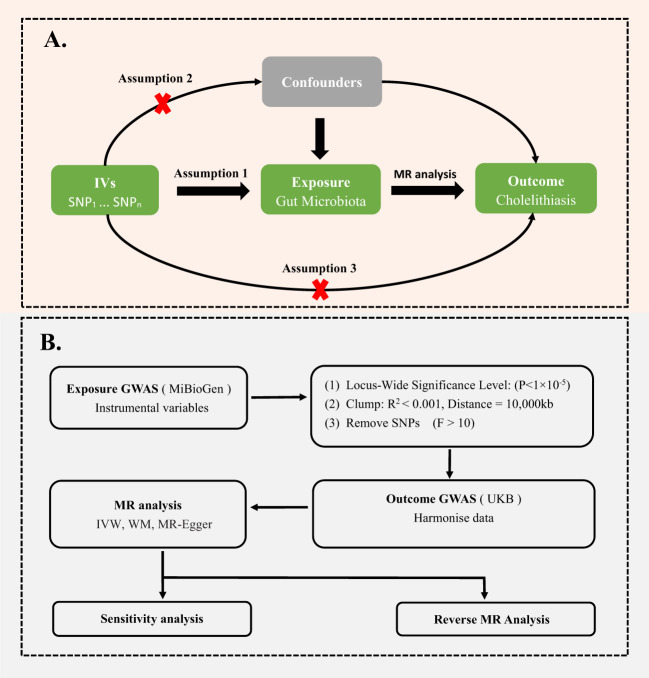
**(A)** Three assumptions of Mendelian randomization. **(B)** Flowchart of this Mendelian randomization study. GWAS, Genome Wide Association Studies; IV, Instrumental variable; SNP, single nucleotide polymorphism; MR, Mendelian randomization; IVW, Inverse-variance weighted; WM, Weighted median; UKB, UK Biobank.

MR analysis is a gene-based method using the random allocation of genetic variants at conception to draw conclusions about the causal effects of exposure on the outcome. To obtain reliable results, as shown in the **Figure 1A**, two-sample MR should satisfy three key assumptions (Bowden and Holmes, 2019): (1) IVs are significantly associated with gut microbiota; (2) IVs are not associated with confounding factors other than gut microbiota; (3) IVs can only affect the cholelithiasis through gut microbiota;.

### 2.2 Data selection

#### 2.2.1 Exposure GWAS: gut microbiota

Summary-level statistical data for gut microbiota was obtained from MiBioGen (data link: https://mibiogen.gcc.rug.nl), which is the largest 16S fecal microbiota data available from 18,340 individuals (24 cohorts from Europe, North America, and East Asia), containing 211 taxa with 122,110 variant sites (Kurilshikov et al., 2021), adjusting for age, sex, study-specific covariates, and the top genetic principal components for population stratification (Kurilshikov et al., 2021).

To ensure the accuracy of the results, we screened the data extracted from MiBioGen. First, since gene loci identified by GWAS for gut microbiota rarely reach genome-wide significance levels (p < 10^-8^), we selected exposure data with p < 10^-5^ to obtain more correlation results (Sanna et al., 2019). Second, to ensure no linkage disequilibrium among gene tools, we chose r^2^ < 0.001 and clumping distance = 10,000kb. Using the following formula, we calculated the *F* and *R^2^* values of each SNP to analyze its impact on gut microbiota: *F = [R^2^ × (N-2)]/(1-R^2^), R^2^ =[2 × β^2^ × EAF × (1-EAF)]/[2 × β^2^ × EAF × (1-EAF) + 2 × SE^2^ × N × EAF × (1-EAF)]* (Burgess and Thompson, 2011; Palmer et al., 2012; Levin et al., 2020). Here, *N* and *EAF* represent the sample size and effect allele frequency, respectively, while *β* and *SE* represent the estimated effect size and standard error of the SNP on gut microbiota (Burgess and Thompson, 2011; Palmer et al., 2012; Levin et al., 2020). We removed SNPs with *F* less than 10, since these SNPs didn’t have sufficient validity (Burgess and Thompson, 2011) (**Figure 1B**).

PhenoScanner V2 (http://www.phenoscanner.medschl.cam.ac.uk/) was used to further assess whether the IVs were potentially associated with confounders or risk factors for cholelithiasis in order to prevent potential pleiotropy. If the IVs had been associated with confounders or risk factors for cholelithiasis, such as body mass index, smoking, or other factors that have been reported, they were excluded from the analysis (**Supplementary Table 6**).

#### 2.2.2 Outcome GWAS: cholelithiasis

The GWAS summary statistics for cholelithiasis were obtained from the UK Biobank, including 6,986 cases and 330,213 controls (http://www.nealelab.is/uk-biobank). The data were adjusted for the first 20 principal components, sex, and age. After obtaining the SNP information for exposure and outcome, we harmonized the data for further analysis.

#### 2.2.3 Reverse MR data

The data source for reverse mendelian randomization is the same as for forward mendelian randomization. In this case, we consider cholelithiasis as the exposure and extract SNPs closely related to cholelithiasis as the exposure (p < 10^-8)^. Similar to forward mendelian randomization, we also conducted a selection process, which included removing linkage disequilibrium and instrument variables with *F* less than 10. We will use significant genera from the forward mendelian randomization analysis as the outcome and then perform a two-sample mendelian randomization analysis to determine the causal relationship between cholelithiasis and gut microbiota.

This MR study was performed using GWAS summary statistics, and ethical approval was obtained by each GWAS. We used published studies and public summary statistics on the website. All of these summary statistics are deidentified, free to download, and be used without limitations.

### 2.3 Data analysis

We used inverse-variance weighted (IVW) as the main MR-analysis method to evaluate the relationships between the human gut microbiome and cholelithiasis (Bowden et al., 2017). The MR-Egger regression was used to test for horizontal pleiotropy. If p>0.05 for MR-Egger intercept, then each SNP satisfies the mendelian hypothesis and the results obtained using IVW are reliable (Bowden et al., 2015), with the potential directional pleiotropy indicated by p<0.05 for MR-Egger intercept. Furthermore, we used MR pleiotropy residual sum and outlier (MR-PRESSO) analysis (Burgess and Thompson, 2011), which identifies and corrects the effects of heterogeneous outliers among the instrument. The Cochrane’s Q test was used to perform the heterogeneity test (Bowden et al., 2017). The leave-one-out sensitivity analysis was performed to verify the presence of unusual instrumental variables that significantly affected the estimation of causal effects (Hemani et al., 2017). Then we performed a reverse mendelian randomization analysis to examine whether a reverse causal association existed between cholelithiasis and gut microbiota. Overall we performed MR analysis and sensitivity analysis in order to obtain reliable GWAS data and credible results.

The MR analysis was performed using the R package “TwoSampleMR”. All statistical analyses and data visualization were performed in R software 4.2.0 (Hemani et al., 2018).

## 3 Results

We utilized the inverse-variance weighted (IVW) method and conducted a sensitivity analysis to find nine gut microbiota taxa with reliable causal relationships with cholelithiasis, as illustrated in **Table 1**. We also provided the results of mendelian randomization analysis of all 211 gut microbiota taxa with gallstone disease in **Supplementary Table 1** and listed the details of all instrumental variables in **Supplementary Table 2**.

**Table 1:** MR results of causal links between gut microbiota and cholelithiasis risk (P < 1×10^-5^).

Classification		Nsnp	SE	P-value	OR (95% CI)	Pleiotropy			Heterogeneity		MR-PRESSO
						Egger intercept	SE	P-value	Q	P-value	
UKB											
Order	*Rhodospirillales*	13	0.001	0.031	0.997 (0.994-0.999)	1.888E-04	5.995E-04	0.759	11.595	0.395	0.467
Genus	*Actinomyces*	5	0.002	0.010	0.995 (0.992-0.999)	-3.058E-04	5.775E-04	0.633	0.761	0.859	0.920
	*Butyrivibrio*	14	0.001	0.032	1.002 (1.001-1.003)	-7.723E-05	5.163E-04	0.884	6.886	0.865	0.906
	*Lachnospiraceae_UCG_001*	13	0.001	0.015	1.003 (1.001-1.006)	6.842E-04	5.715E-04	0.256	6.539	0.835	0.820
	*Phascolarctobacterium*	10	0.002	0.036	0.996 (0.993-0.999)	6.218E-04	6.944E-04	0.397	4.492	0.810	0.811
	*Rikenellaceae_RC9_gutgroup*	10	0.001	0.023	0.998 (0.996-0.999)	4.355E-04	8.043E-04	0.603	7.039	0.532	0.641
	*Ruminococcaceae_NK4A214_group*	14	0.002	0.003	1.005 (1.002-1.009)	-2.255E-05	4.925E-04	0.964	15.167	0.232	0.333
	*Ruminococcaceae_UCG_011*	7	0.001	0.010	1.003 (1.001-1.005)	7.875E-04	7.753E-04	0.356	4.374	0.497	0.531
	*Ruminococcaceae_UCG_013*	11	0.002	0.022	0.996 (0.992-0.999)	-1.053E-05	3.993E-04	0.980	10.982	0.277	0.416

SNP, single nucleotide polymorphism; OR, odds ratio; MR-PRESSO, Mendelian randomization pleiotropy residual sum and outlier; Q, Cochran’s Q.

**Figure 2:**
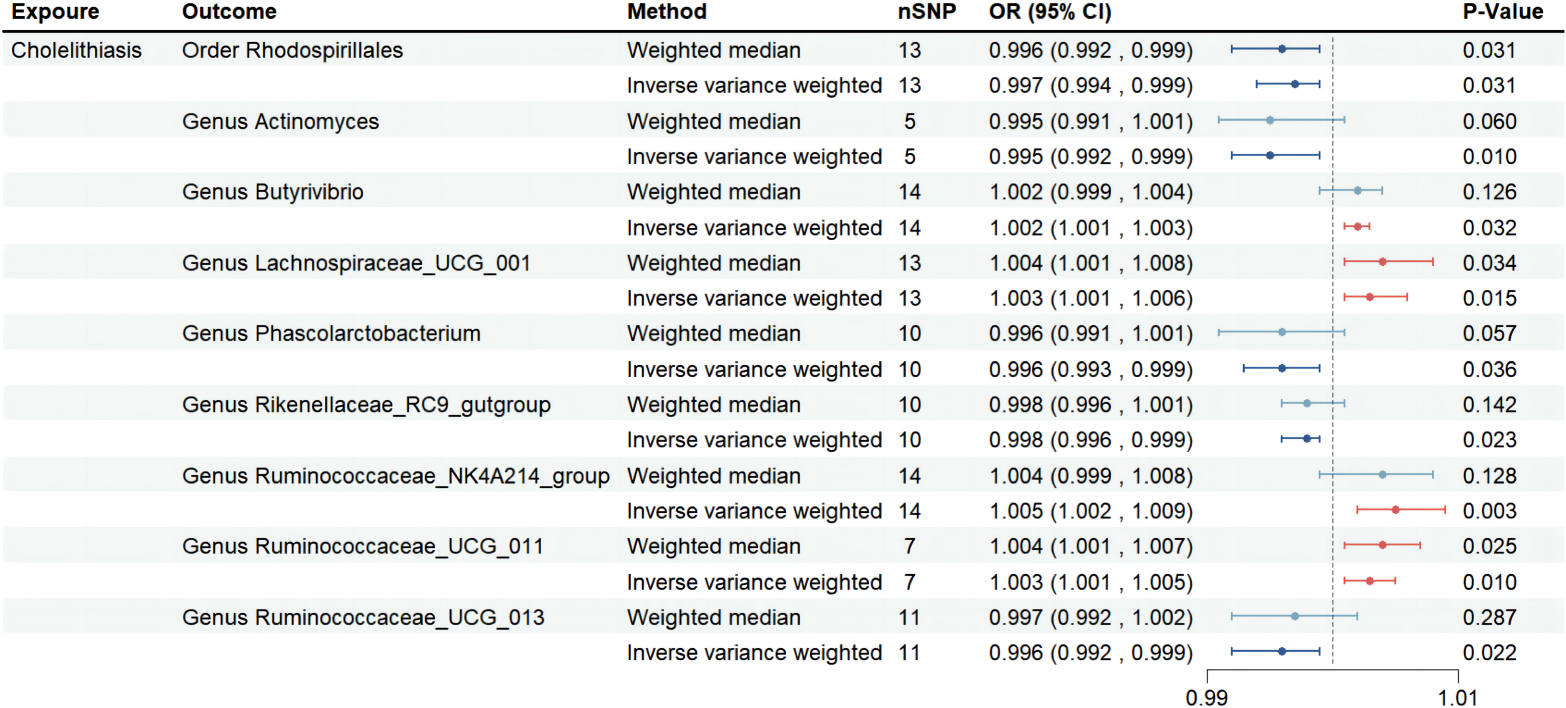
Forest plot of the associations between genetically determined 9 gut microbial genera with the risks of cholelithiasis in UKB. Abbreviations: OR, odds ratio; SNP, single-nucleotide polymorphism; SE, Standard Error.

We identified a positive association between the risk of cholelithiasis and four gut microbiota taxa: *Genus Lachnospiraceae_UCG_001* (OR=1.003, 95%CI:1.001-1.006, p=0.015), *Genus Butyrivibrio* (OR=1.002, 95%CI:1.001-1.003, p=0.032), *Genus Ruminococcaceae_NK4A214_group* (OR=1.005, 95%CI:1.002-1.009, p=0.003), and *Genus Ruminococcaceae_UCG_011* (OR=1.003, 95%CI:1.001-1.005, p=0.010). This suggests that these bacteria may increase the risk of cholelithiasis. Sensitivity analysis did not reveal any evidence of horizontal pleiotropy. Weighted median analysis was performed on four gut microbiota taxa, and the directionality obtained in the forest plot was consistent with IVW (**Figure 2**).

On the other hand, we found that five gut microbiota taxa were associated with a reduced risk of cholelithiasis: *Order Rhodospirillales* (OR=0.997, 95%CI: 0.994-0.999, p=0.031), *Genus Actinomyces* (OR=0.995, 95%CI: 0.992-0.999, p=0.010), *Genus Phascolarctobacterium* (OR=0.996, 95%CI: 0.993-0.999, p=0.036), *Genus Rikenellaceae_RC9_gutgroup* (OR=0.998, 95%CI: 0.996-0.999, p=0.023), and *Genus Ruminococcaceae_UCG_013* (OR=0.996, 95%CI: 0.992-0.999, p=0.022). This suggests that these bacteria may have a protective effect against cholelithiasis. Sensitivity analysis did not reveal any evidence of horizontal pleiotropy. Weighted median analysis was performed on five gut microbiota taxa, and the directionality obtained in the forest plot was consistent with IVW (**Figure 2**). No abnormal SNP was found in the Leave-one-out test. The scatter plot and the results of the Leave-one-out test are shown in the **supplementary figure**. In conclusion, the above results demonstrate the existence of a stable causal relationship between gut microbiota and cholelithiasis based on genetics.

## 4 Reverse MR analysis

We conducted a reverse mendelian randomization analysis using the IVW method to investigate the causal relationship between nine gut microbiota taxa and cholelithiasis. After removing linkage disequilibrium, we obtained 10 SNPs that were strongly associated with cholelithiasis, with each SNP having an *F* greater than 10.

As shown in **Table 2**, none of the gut microbiota taxa showed a significant reverse causal relationship with cholelithiasis in the UKB dataset, including *Order Rhodospirillales* (p=0.659), *Genus Actinomyces* (p=0.590), *Genus Butyrivibrio* (p=0.880), *Genus Lachnospiraceae_UCG_001* (p=0.422), *Genus Phascolarctobacterium* (p=0.119), *Genus Rikenellaceae_RC9_gutgroup* (p=0.806), *Genus Ruminococcaceae_NK4A214_group* (p=0.681), *Genus Ruminococcaceae_UCG_011* (p=0.228), and *Genus Ruminococcaceae_UCG_013* (p=0.954). Our MR-Egger regression method and Cochrane’s Q test also confirmed the reliability of our results.

**Table 2:** MR results of causal links between cholelithiasis and gut microbiota risk.

Classification		Nsnp	SE	P-value	OR (95% CI)	Pleiotropy			Heterogeneity	
						Egger intercept	SE	P-value	Q	P-value
UKB										
Order	*Rhodospirillales*	10	1.413	0.659	1.865(0.1169-29.7742)	-7.982E-03	8.957E-03	0.399	9.999	0.351
Genus	*Actinomyces*	10	1.450	0.590	2.182(0.1273-37.4101)	1.121E-02	1.544E-02	0.488	2.551	0.979
	*Butyrivibrio*	10	2.413	0.880	1.438(0.0127-162.8934)	1.210E-02	7.699E-03	0.155	12.063	0.210
	*Lachnospiraceae_UCG_001*	10	1.247	0.422	0.367(0.0319-4.2360)	7.595E-04	7.350E-03	0.920	6.907	0.647
	*Phascolarctobacterium*	10	1.188	0.119	0.157(0.0153-1.6066)	1.279E-02	7.777E-02	0.874	5.845	0.755
	*Rikenellaceae_RC9_gutgroup*	9	4.579	0.806	3.085(0.0004-24387.7118)	-7.014E-03	9.765E-03	0.493	13.606	0.093
	*Ruminococcaceae_NK4A214_group*	10	1.537	0.681	0.531(0.0261-10.8081)	7.639E-03	9.016E-03	0.421	20.650	0.014
	*Ruminococcaceae_UCG_011*	10	1.435	0.228	5.635(0.3387-93.7602)	6.133E-03	6.004E-03	0.337	15.221	0.085
	*Ruminococcaceae_UCG_013*	10	0.974	0.954	1.058(0.1569-7.1315)	5.352E-03	9.101E-03	0.573	6.500	0.689

SNP, single nucleotide polymorphism; OR, odds ratio; MR-PRESSO, Mendelian randomization pleiotropy residual sum and outlier; Q, Cochran’s Q.

## 5 Discussion

To our knowledge, this is the first mendelian randomization study to assess the causal role of gut microbiota on cholelithiasis. Our results suggest that specific gut microbiota is causally associated with cholelithiasis.

The role of gut microbiota in the development of gallstone disease has been extensively studied, with many studies highlighting the correlation between them. For instance, Wang et al. used 16S rRNA gene sequencing to investigate changes in the composition of the gut microbiota in mice fed a lithogenic diet. They found that the abundance and diversity of gut microbiota were significantly reduced in mice fed a lithogenic diet compared to the control group. Moreover, the ratio of *Firmicutes/Bacteroidetes* and the *Firmicutes* content were reduced as well, indicating the potential impact of gut microbiota on the formation of gallstones (Wang et al., 2017). Similarly, Keren et al. showed that gallstone patients had a higher overall concentration of fecal bile acids (BAs) and a decreased microbial diversity, which was accompanied by a reduction in the beneficial *Genus Roseburia* and an enrichment of the uncultivated *Genus Oscillospira*, compared with the control group (Keren et al., 2015). These findings suggest that gut microbiota dysbiosis, as characterized by a reduction in microbial diversity and alterations in specific bacterial taxa, may be associated with an increased risk of gallstone disease. These studies provide important insights into the potential role of gut microbiota in the development of gallstone disease. Further research is needed to better understand the underlying mechanisms and to identify potential therapeutic targets for the prevention and treatment of this common condition.

The pathogenesis of cholelithiasis is complex and closely related to metabolism (Lammert et al., 2016). Cholesterol stones account for more than 80% of cholelithiasis (Lammert et al., 2016). The state of biliary cholesterol supersaturation, increased ability to crystallize cholesterol in the bile and the dysfunction of gallbladder motility is currently recognized as the pathophysiological basis for cholelithiasis formation (Di Ciaula et al., 2018). Disturbances in bile acid metabolism are thought to be a key step in gallbladder stone formation, gut microbiota may further influence gallstone formation by regulating the hepatic-intestinal circulation of bile acids (Hu et al., 2022). Therefore, it is important to explore the relationship between cholelithiasis from the perspective of gut microbiota and bile acids.

Our results suggested that *Genus Lachnospiraceae_UCG001* was positively causal to cholelithiasis risk. The *Lachnospiraceae* is known to have a *7 α -dehydroxylated* activity, and increased *7α-dehydroxylase* activity can promote the formation of cholesterol stones (Ridlon et al., 2016). Secondary bile acids are regulated by *7α-dehydroxylase* activity, and the increased activity of this enzyme increases the production of secondary bile acids (Kriaa et al., 2019). However, the body itself cannot effectively remove secondary bile acids by metabolism, and therefore these secondary bile acids accumulate to high levels in the bile, increasing the potential for cholesterol stone formation (Hu et al., 2022).

Butyric acid, one of the major members of the short-chain fatty acids, is produced in the intestine mainly by the enzymatic digestion of dietary fiber and is used as the main energy source for the intestinal epithelium (Ye et al., 2021). Butyrate-producing bacteria include *Ruminococcaceae* (Su et al., 2022), *Butyrivibrio*, etc., which increase the content of butyrate in the intestine, enhancing the activity of bile salt hydrolase (BSH) and increasing free bile acids in the intestinal lumen, while hydrophobic free bile acids are not easily reabsorbed by the intestine and are excreted in the feces (Wang et al., 2012; Ye et al., 2021). To compensate for the loss of bile acids, the liver uses the cholesterol in the blood as a raw material to synthesize bile acids, thus speeding up the process of converting cholesterol into bile acids, resulting in lower cholesterol in the blood and reducing the formation of gallbladder stone (Wang et al., 2012; Ye et al., 2021). Our study showed that *Genus Butyrivibrio* (OR=1.002), *Genus Ruminococcaceae_NK4A214_group* (OR=1*.005), Genus Ruminococcaceae_UCG_011* (OR=1.003) and *Genus Ruminococcaceae_UCG-010* (OR=0.997) had opposite effects on cholelithiasis, which provides a new perspective for future studies.

We found no further studies on the association between *Phascolarctobacterium* and cholelithiasis, but one study showed fecal taurine-conjugated chenodeoxycholic acid correlated with *Phascolarctobacterium* (Yang et al., 2022), which may inspire future studies on *Phascolarctobacterium* and cholelithiasis. As there are few studies on specific flora and gallstone disease, we found a limited number of relevant studies on several other florae. In addition, to our knowledge, we first time reported that *Order Rhodospirillales*, *Genus Rikenellaceae_RC9_gutgroup* and *Genus_Actinomyces* are associated with the risk of cholelithiasis, which may provide new directions for subsequent studies.

Overall, our study has several innovative points: first, current studies have mostly focused on the correlation analysis at the family level, we further analyzed the causality of specific gut microbiota for cholelithiasis at 5 levels from genus to phylum and identified gut microbiota that may have an impact on cholelithiasis; Secondly, compared to previous randomized controlled studies, our mendelian randomization study based on GWAS has a larger sample size. In addition, we conducted a reverse mendelian randomization study and did not find reverse causality. Of course, there are certain limitations to our study. Although our study satisfies the MR hypothesis, it still does not guarantee weak instrumental bias. As most of the subjects included in the study were of European origin, the results of this study may not be generalizable to other ethnic groups.

## 6 Conclusions

Our findings suggest that specific gut microbiota can influence cholelithiasis. Several types of gut microbiota identified in this study may influence the development of cholelithiasis and provide new directions for the future prevention and treatment of cholelithiasis.

## Data availability statement

The datasets presented in this study can be found in online repositories. The names of the repository/repositories and accession number(s) can be found in the article/**Supplementary Material**.

## Author contributions

SL and WL designed the study. WL performed the main data analysis and wrote the draft of the manuscript. QQ, AR, and LZ conducted the data acquisition and performed the data analysis and manuscript revision. Both QP and RM contributed to the data analysis and manuscript revision. SL supervised the whole research and is responsible for the integrity of data analysis. All authors contributed to the article and approved the submitted version.
