# Supplementary material for: Causal associations between human gut microbiota and cholelithiasis: a mendelian randomization study
Source: Front Cell Infect Microbiol. 2023 May 25;13:1169119. doi: 10.3389/fcimb.2023.1169119 (PMC10248444; doi:10.3389/fcimb.2023.1169119)
Supplement: Supplementary file 1 [file DataSheet_1.pdf]

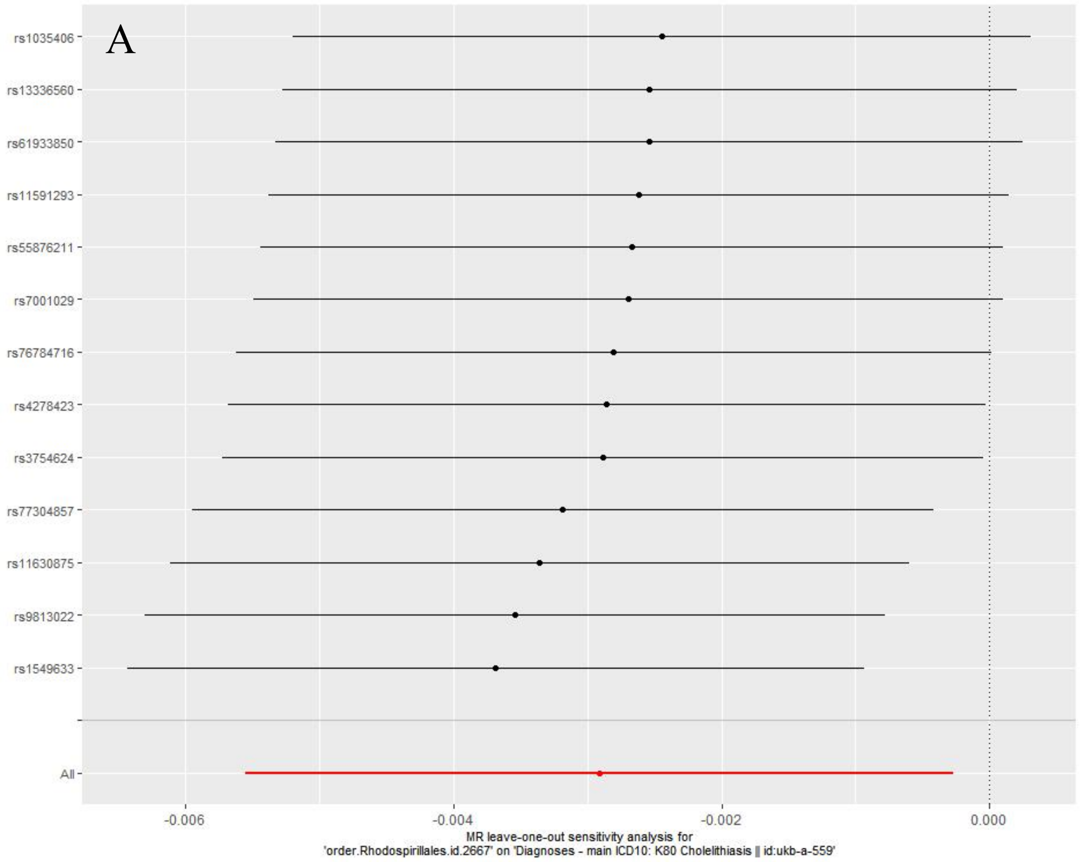

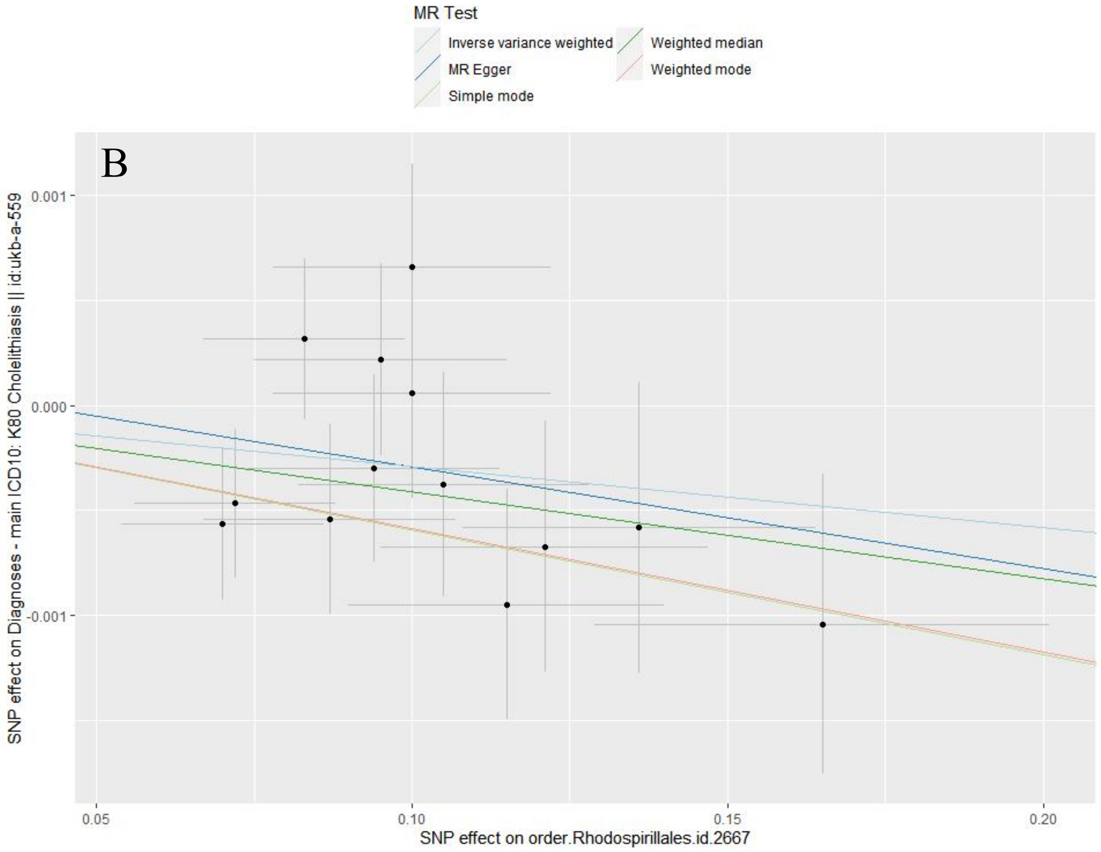

Figure S1. (A) Leave-one-out sensitivity analysis for *Order Rhodospirillales* on Cholelithiasis. (B) Scatter plots for effect sizes of SNPs for *Order Rhodospirillales* on Cholelithiasis. Abbreviations: SNP, single nucleotide polymorphism; MR-PRESSO, Mendelian randomization.

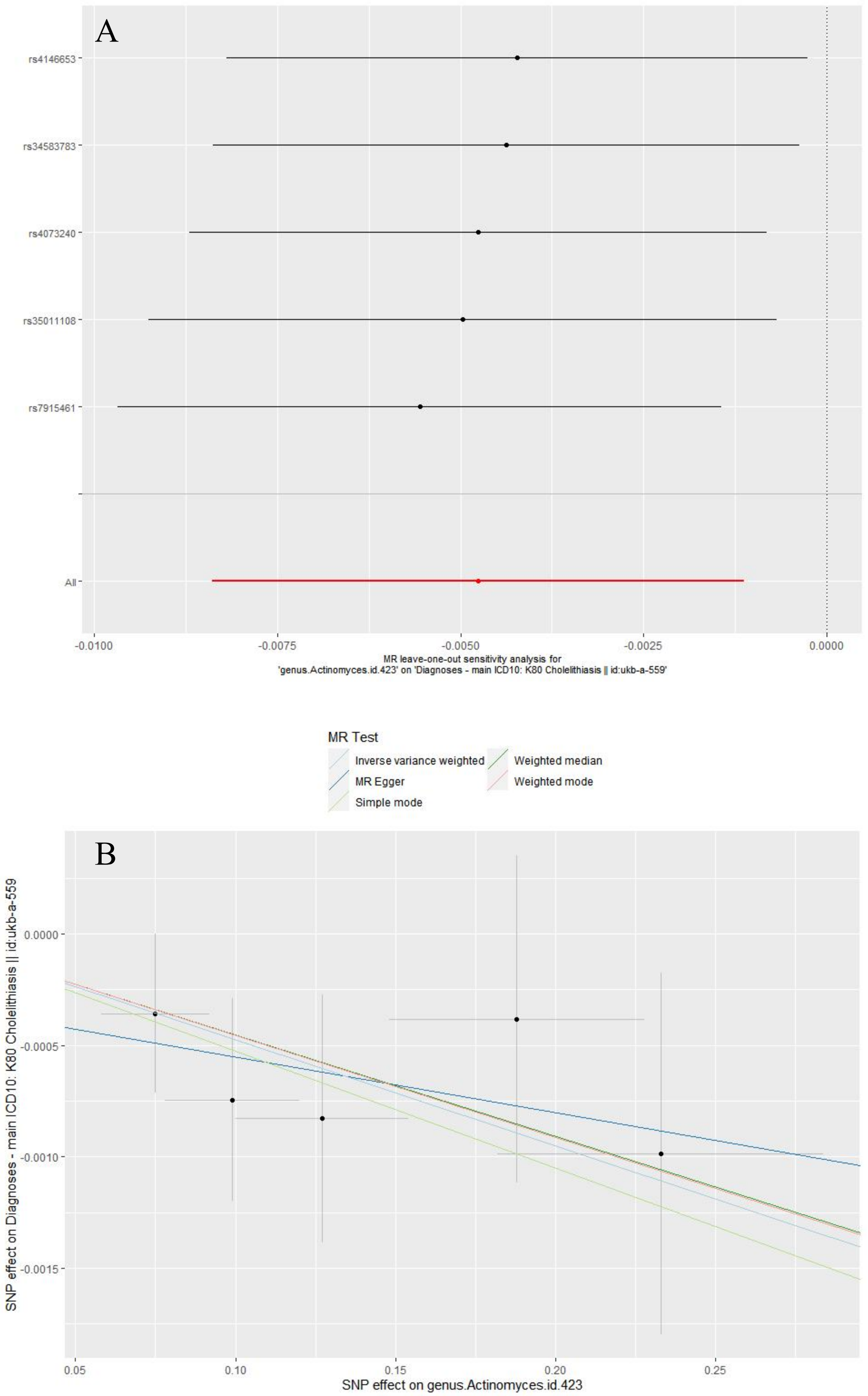

Figure S2. (A) Leave-one-out sensitivity analysis for *Genus Actinomyces* on Cholelithiasis. (B) Scatter plots for effect sizes of SNPs for *Genus Actinomyces* on Cholelithiasis. Abbreviations: SNP, single nucleotide polymorphism; MR-PRESSO, Mendelian randomization.

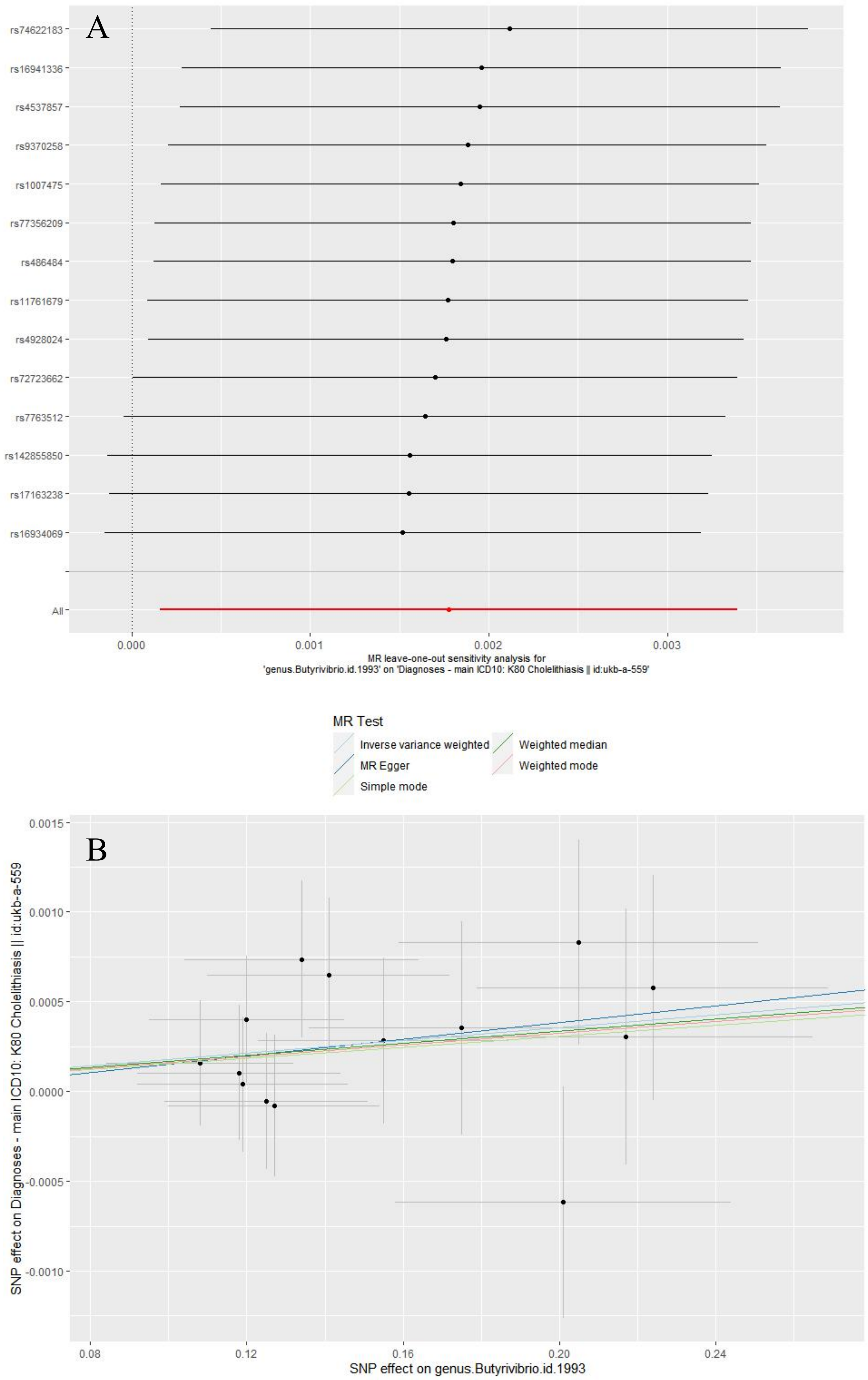

Figure S3. (A) Leave-one-out sensitivity analysis for *Genus Butyrivibrio* on Cholelithiasis. (B) Scatter plots for effect sizes of SNPs for *Genus Butyrivibrio* on Cholelithiasis. Abbreviations: SNP, single nucleotide polymorphism; MR-PRESSO, Mendelian randomization.

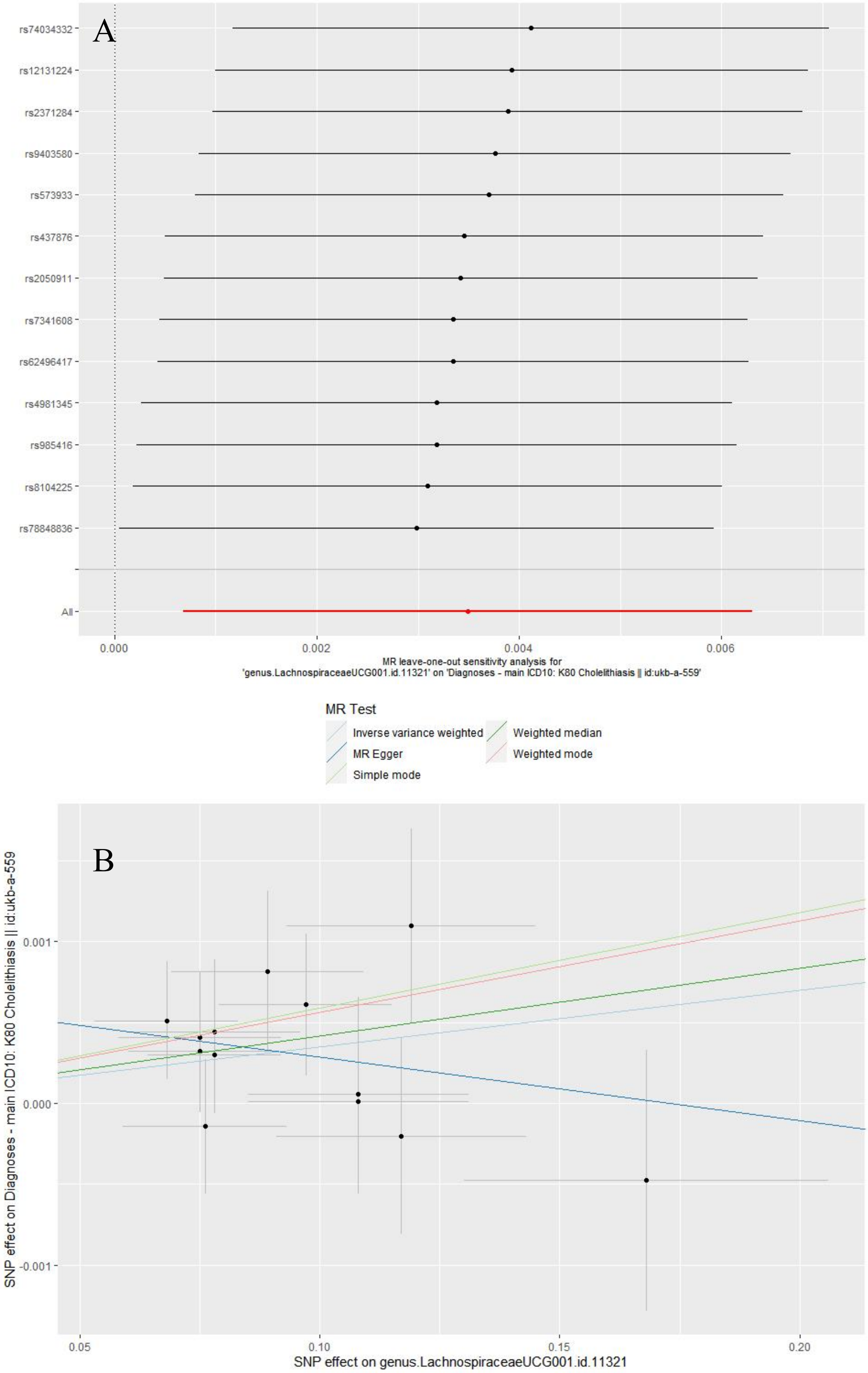

Figure S4. (A) Leave-one-out sensitivity analysis for *Genus Lachnospiraceae_UCG_001* on Cholelithiasis. (B) Scatter plots for effect sizes of SNPs for *Genus Lachnospiraceae_UCG_001* on Cholelithiasis. Abbreviations: SNP, single nucleotide polymorphism; MR-PRESSO, Mendelian randomization.

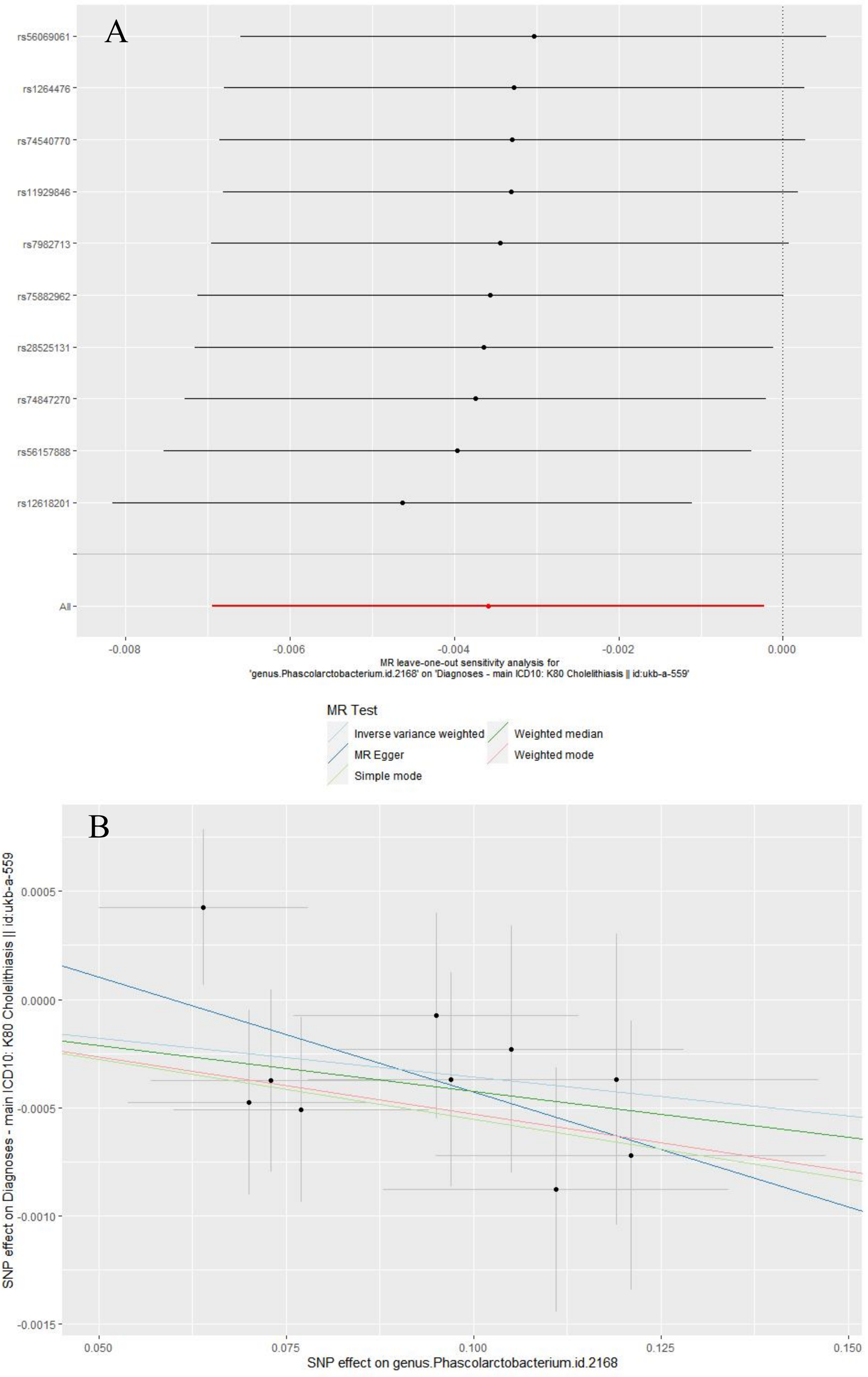

Figure S5. (A) Leave-one-out sensitivity analysis for *Genus Phascolarctobacterium* on Cholelithiasis. (B) Scatter plots for effect sizes of SNPs for *Genus Phascolarctobacterium* on Cholelithiasis. Abbreviations: SNP, single nucleotide polymorphism; MR-PRESSO, Mendelian randomization.

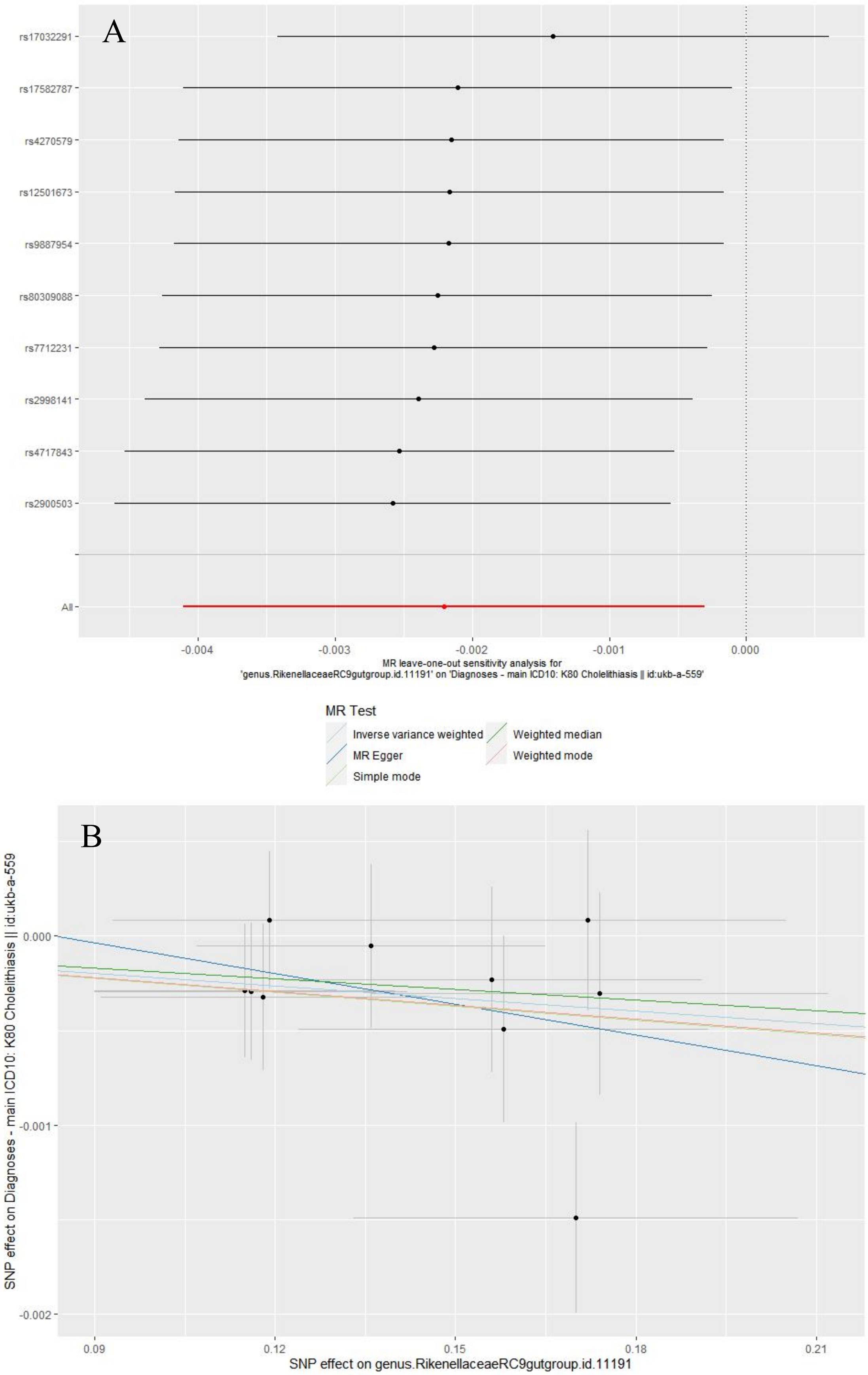

Figure S6. (A) Leave-one-out sensitivity analysis for *Genus Rikenellaceae_RC9_gutgroup* on Cholelithiasis. (B) Scatter plots for effect sizes of SNPs for *Genus Rikenellaceae_RC9_gutgroup* on Cholelithiasis. Abbreviations: SNP, single nucleotide polymorphism; MR-PRESSO, Mendelian randomization.

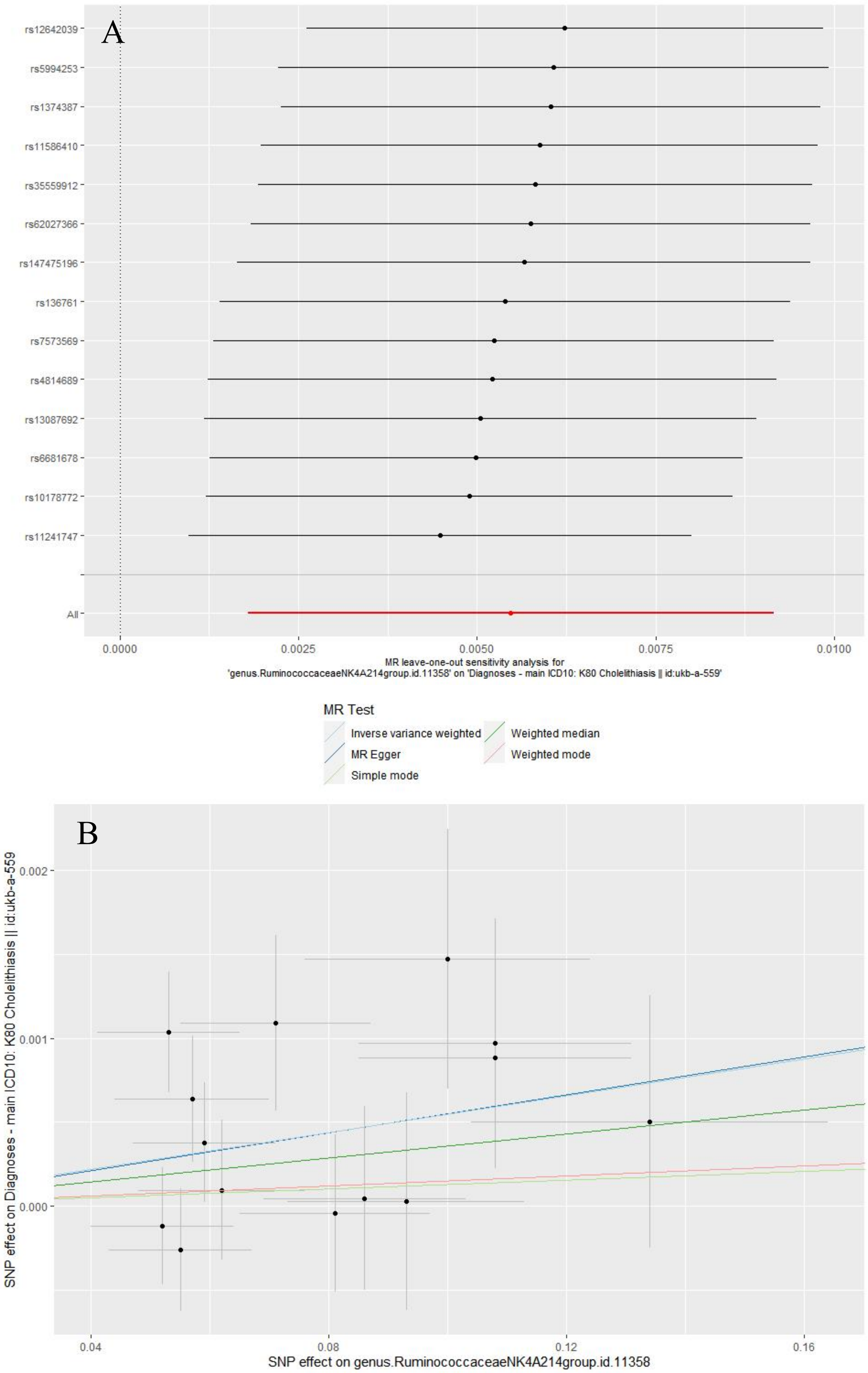

Figure S7. (A) Leave-one-out sensitivity analysis for *Genus Ruminococcaceae_NK4A214_group* on Cholelithiasis. (B) Scatter plots for effect sizes of SNPs for *Genus Ruminococcaceae_NK4A214_group* on Cholelithiasis. Abbreviations: SNP, single nucleotide polymorphism; MR-PRESSO, Mendelian randomization.

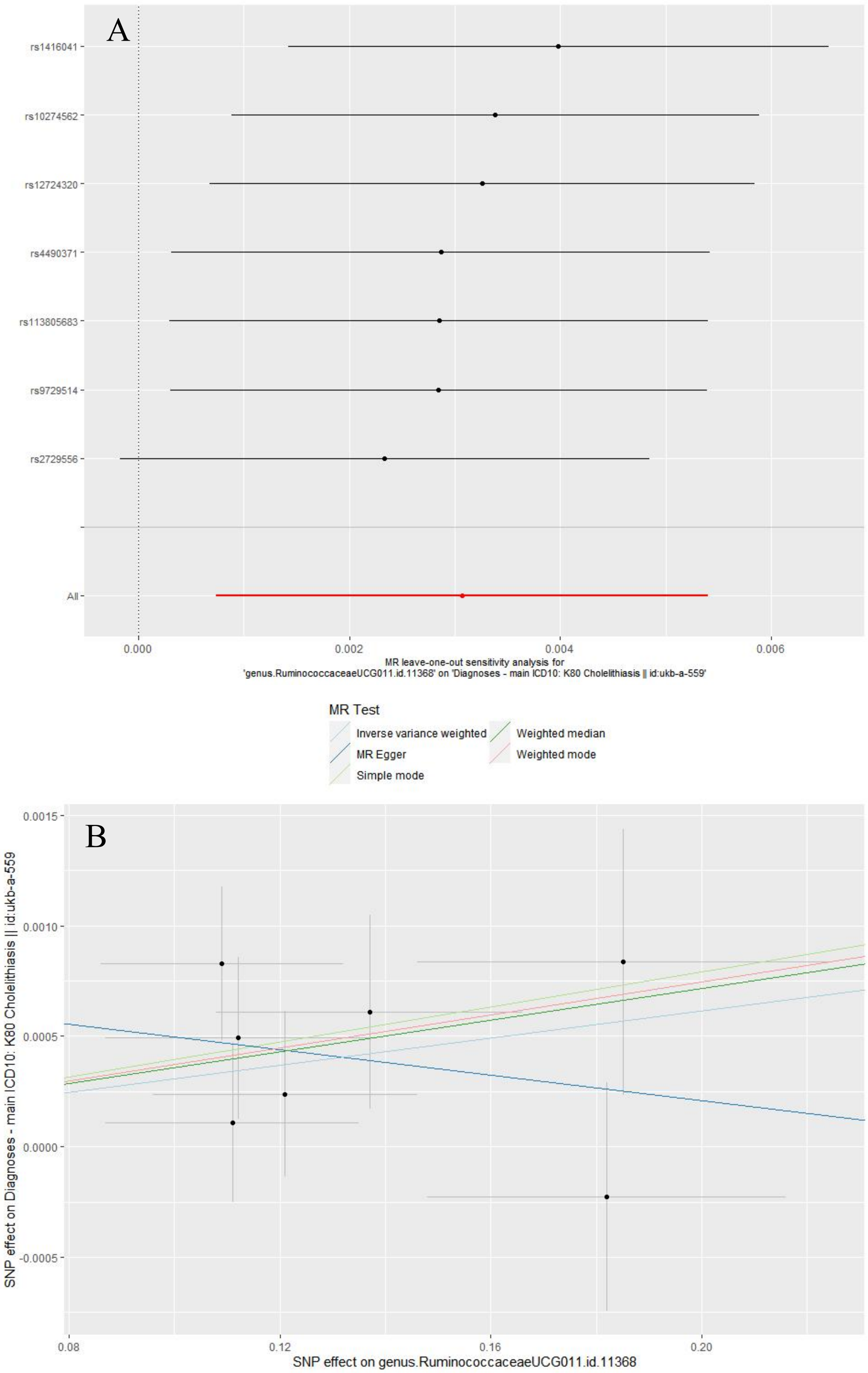

Figure S8. (A) Leave-one-out sensitivity analysis for *Genus Ruminococcaceae_UCG_011* on Cholelithiasis. (B) Scatter plots for effect sizes of SNPs for *Genus Ruminococcaceae_UCG_011* on Cholelithiasis. Abbreviations: SNP, single nucleotide polymorphism; MR-PRESSO, Mendelian randomization.

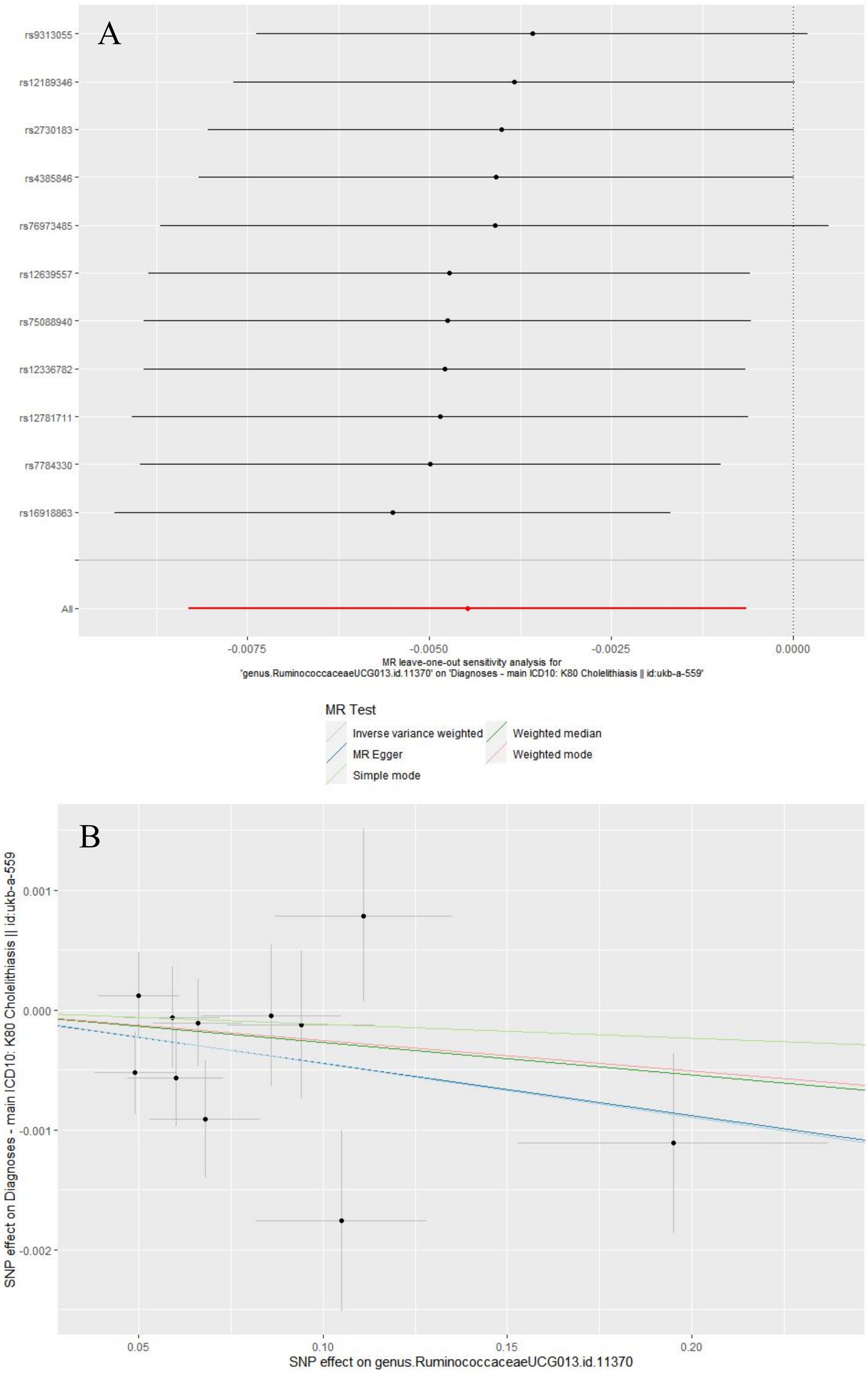

Figure S9. (A) Leave-one-out sensitivity analysis for *Genus Ruminococcaceae_UCG_013* on Cholelithiasis. (B) Scatter plots for effect sizes of SNPs for *Genus Ruminococcaceae_UCG_013* on Cholelithiasis. Abbreviations: SNP, single nucleotide polymorphism; MR-PRESSO, Mendelian randomization.
